# Hypothermic Oxygenated Machine Perfusion Prevents Arteriolonecrosis of the Peribiliary Plexus in Pig Livers Donated after Circulatory Death

**DOI:** 10.1371/journal.pone.0088521

**Published:** 2014-02-14

**Authors:** Sanna op den Dries, Michael E. Sutton, Negin Karimian, Marieke T. de Boer, Janneke Wiersema-Buist, Annette S. H. Gouw, Henri G. D. Leuvenink, Ton Lisman, Robert J. Porte

**Affiliations:** 1 Section of Hepatobiliary Surgery and Liver Transplantation, Department of Surgery, University of Groningen, University Medical Center Groningen, Groningen, The Netherlands; 2 Surgical Research Laboratory, University of Groningen, University Medical Center Groningen, Groningen, The Netherlands; 3 Department of Pathology, University of Groningen, University Medical Center Groningen, Groningen, The Netherlands; university of birmingham, United Kingdom

## Abstract

**Background:**

Livers derived from donation after circulatory death (DCD) are increasingly accepted for transplantation. However, DCD livers suffer additional donor warm ischemia, leading to biliary injury and more biliary complications after transplantation. It is unknown whether oxygenated machine perfusion results in better preservation of biliary epithelium and the peribiliary vasculature. We compared oxygenated hypothermic machine perfusion (HMP) with static cold storage (SCS) in a porcine DCD model.

**Methods:**

After 30 min of cardiac arrest, livers were perfused *in situ* with HTK solution (4°C) and preserved for 4 h by either SCS (n = 9) or oxygenated HMP (10°C; n = 9), using pressure-controlled arterial and portal venous perfusion. To simulate transplantation, livers were reperfused *ex vivo* at 37°C with oxygenated autologous blood. Bile duct injury and function were determined by biochemical and molecular markers, and a systematic histological scoring system.

**Results:**

After reperfusion, arterial flow was higher in the HMP group, compared to SCS (251±28 vs 166±28 mL/min, respectively, after 1 hour of reperfusion; p = 0.003). Release of hepatocellular enzymes was significantly higher in the SCS group. Markers of biliary epithelial injury (biliary LDH, gamma-GT) and function (biliary pH and bicarbonate, and biliary transporter expression) were similar in the two groups. However, histology of bile ducts revealed significantly less arteriolonecrosis of the peribiliary vascular plexus in HMP preserved livers (>50% arteriolonecrosis was observed in 7 bile ducts of the SCS preserved livers versus only 1 bile duct of the HMP preserved livers; p = 0.024).

**Conclusions:**

Oxygenated HMP prevents arteriolonecrosis of the peribiliary vascular plexus of the bile ducts of DCD pig livers and results in higher arterial flow after reperfusion. Together this may contribute to better perfusion of the bile ducts, providing a potential advantage in the post-ischemic recovery of bile ducts.

## Introduction

Livers from donation after circulatory death (DCD) donors are increasingly used for transplantation. Although this may help to increase the number of donor livers available for transplantation, DCD livers are associated with a higher risk of nonanastomotic biliary strictures (NAS), compared to livers donated after brain death (DBD) [1]–[4]. NAS have been reported in 20–33% of patients receiving a DCD liver, compared with 0–13% of patients receiving a DBD liver graft, consequently contributing to a higher rate of morbidity and graft failure after DCD liver transplantation [5]–[7]. In contrast to DBD liver grafts, livers from DCD donors suffer from warm ischemia in the donor during the time period between cardiac arrest and *in situ* cold perfusion. This donor warm ischemia in combination with subsequent cold ischemia during organ preservation is believed to be a main cause of bile duct injury leading to NAS after transplantation [8]. Biliary epithelial cells have been shown to be more susceptible to warm ischemic injury than hepatocytes, which may explain the high rate of NAS following otherwise successful DCD liver transplantation [9], [10]. In a recent clinical study, using a standardized histological evaluation of biopsies taken from the extrahepatic bile duct at the time of transplantation, arteriolonecrosis of the peribiliary vascular plexus was identified as the only independent histological parameter predictive for the development of NAS after transplantation [11].

Machine perfusion is increasingly discussed as a promising tool to optimize livers before transplantation. During machine preservation livers are perfused with an oxygenated or non-oxygenated perfusion fluid at either low temperature or normal body temperature [12]–[16]. So far, most investigations have focused on hypothermic machine perfusion (HMP) and studies have suggested that oxygenated HMP results in better preservation of the liver parenchyma, compared to the classical method of organ preservation, static cold storage (SCS) [14]–[16]. Although machine perfusion may be particularly beneficial for preservation of hepatocellular energy status and viability of high-risk liver grafts, it is still unclear whether it also provides better protection of the biliary epithelium and the peribiliary vascular plexus, especially in livers from DCD donors.

We hypothesized that oxygenated HMP leads to reduced injury of the biliary epithelium and the bile duct wall, compared to SCS. To test this hypothesis we compared oxygenated HMP and SCS in an established DCD model of liver donation in pigs, followed by *ex vivo* reperfusion using oxygenated autologous blood.

## Materials and Methods

### Liver Procurement and Preservation

Experiments were performed in accordance with the Dutch Law on Animal Experiments and the study protocol was approved by the Institutional Animal Care and Use Committee of the University of Groningen (DEC 6411).

Dutch Landrace pigs (n = 18; age 4–5 months; weight 90–110 kg) were premedicated with atropine, tiletamine/zolazepam, and Finadyne. General anaesthesia was induced with a 4% sevoflurane mixture with air/oxygen and maintained after intubation by mechanical ventilation using 2% sevoflurane. After administration of 20,000 IU heparin and 4 mg pancuronium iv mechanical ventilation was stopped and cardiac arrest was awaited. Animals were subsequently left untouched for 30 min, resulting in a mean time interval between cessation of ventilation and *in situ* cooling of 48 min. A midline laparotomy was performed and the distal aorta and inferior vena cava were cannulated for rapid *in situ* flush out with ice-cold histidine-tryptophan-ketoglutarate (HTK) preservation solution. The abdominal cavity was filled with slush ice for additional topical cooling. The first 2L of blood, drained from the inferior vena cava, was collected in a polyethylene bag with 20.000 IU of heparin.

On the backtable, liver grafts were perfused with an additional 1L of cold HTK via the portal vein, and the cystic duct was ligated. After careful flushing of the bile duct with preservation solution, an 8Fr silicon catheter was inserted and secured in the distal extrahepatic bile duct for collection of bile. The mean liver mass at the start of the experiment was 1365±140 grams.

Livers were randomly assigned to one of the following two groups: SCS for 4 h, followed by 2 h of *ex vivo* reperfusion with autologous blood at 37°C (n = 9) or oxygenated HMP for 4 h (n = 9), followed by 2 h of *ex vivo* reperfusion with autologous blood at 37°C.

### Hypothermic Oxygenated Machine Perfusion

HMP was initiated immediately after procurement, using a CE marked device that enables dual perfusion via both the hepatic artery and the portal vein in a closed circuit (Liver Assist®, Organ Assist, Groningen, Netherlands). Livers were perfused for 4 h with Belzer machine perfusion fluid (Bridge-to-Life, Ltd., Northbrook, IL), a UW based perfusion fluid, oxygenated with 100% O_2_. Two rotary pumps provided pulsatile flow to the hepatic artery and continuous flow to the portal vein. The flows during 4 hours of HMP are presented in *Figure S1*. Two hollow fiber membrane oxygenators provided oxygenation of the perfusion solution and removal of CO_2_. The system was both pressure and temperature (10°C) controlled, allowing auto-regulation of the blood flow through the liver. Pressure was limited to a mean of 30 mmHg in the hepatic artery and 5 mmHg in the portal vein.

Livers that were preserved by SCS were packed in sterile organ bags with ice-cold HTK preservation fluid on melting ice.

### Normothermic Reperfusion

To simulate transplantation, liver grafts were reperfused with pig blood, using the same perfusion machine as used for HMP. Temperature was kept at 37°C and mean arterial and portal pressures were limited to 60 mmHg and 11 mmHg, respectively. Flow was monitored continuously. Sodium bicarbonate (8.4% solution) was added to the perfusion fluid to maintain a physiological pH.

### Assessment of Hepatocellular Function and Injury

During graft reperfusion, samples were taken from the perfusion fluid every 30 min and analyzed immediately for blood gas parameters (pO_2_, pCO_2_, sO_2_, HCO_3_^–^ and pH) using an ABL800 FLEX analyzer (Radiometer, Brønshøj, Denmark). In addition, plasma from the perfusion fluid was collected, frozen and stored at –80°C for determination of alkaline phosphatase, gamma-glutamyl transferase (gamma-GT), aspartate aminotransferase (AST), lactate dehydrogenase (LDH), and total bilirubin, using standard biochemical methods (clinical grade). Total biliary bile salt concentration was measured spectrophotometrically using 3-hydroxysteroid dehydrogenase [17]. Biliary phospholipid concentration was analyzed using a commercially available enzymatic method; the Phospholipids FS Kit (DiaSys Diagnostic Systems, Holzheim, Germany).

### Assessment of Biliary Epithelial Cell Function and Injury

After reperfusion, bile production was measured gravimetrically (in grams) at 30 min intervals. Biliary epithelial cell function was assessed by measuring pH, bicarbonate and glucose concentration in bile. For this purpose, bile samples were collected under mineral oil and analyzed immediately using an ABL800 FLEX analyzer. In addition, biliary concentrations of gamma-GT, alkaline phosphatase, and LDH were measured as biomarkers of biliary epithelial injury [18]. Thiobarbituric acid reactive substances (TBARS) were measured in bile samples as a marker for oxidative stress in bile ducts (*see File S1*).

### Gene Expression of Hepatobiliary Transporter Proteins

Hepatic mRNA expression of relevant hepatocellular and cholangiocyte transporter proteins involved in bile secretion was determined by quantitative real-time PCR as described previously [19]. Sense and antisense porcine-specific primers used for real-time PCR as well as a detailed description of the methods can been found in *File S1 and Table S1*.

### Adenosine-5'-triphosphate (ATP) Extraction and Measurement

Hepatic concentration of ATP was used as an indicator of the energy status of grafts. Liver samples were immediately frozen in liquid nitrogen and processed for quantification of ATP as described in the *File S1*.

### Histological Evaluation of Liver Parenchyma and Bile Ducts

Before and after SCS or HMP, biopsies were taken from liver parenchyma and distal extrahepatic bile ducts. After reperfusion, additional biopsies were taken from liver parenchyma, the extrahepatic bile duct (proximal from the tip of the biliary catheter), and from the intrahepatic bile ducts beyond the level of the first and second bifurcation. Bile ducts were gently grasped with fine forceps, taking care not to touch the mucosa and specimens were excised using fine Metzenbaum scissors or a scalpel. Biopsies were divided into two sections: one preserved in 10% formaldehyde for inclusion in paraffin and one snap-frozen in liquid nitrogen and stored at −80°C for RNA extraction. Paraffin-embedded slides were prepared for hematoxylin and eosin (H&E) staining. Additional slides were prepared for immunohistochemical detection of activated caspase-3 (Asp175, Cell Signaling #9661; 1∶100), a marker for apoptosis. Injury of bile ducts was semiquantified using a systematic scoring system as previously described by Hansen et al. [11] for human donor livers. In particular, arteriolonecrosis was defined by complete loss of vital cells in the wall of arterioles and small arteries, which are commonly arranged around the bile duct wall [11]. All sections were examined in a blinded fashion by two independent observers. In case of discordant results slides were examined by a third investigator.

### Statistical Analysis

Continuous variables are presented as mean ± standard error (SE). Categorical variables are presented as number and percentage. Continuous variables were tested for normality using a Kolmogorov-Smirnov test and comparisons between groups were performed using the Student-T test for normally distributed variables and the Mann-Whitney test for not normally distributed variables. Comparisons within groups were performed by a paired T-test or Wilcoxon test as appropriate. Categorical variables were compared with the Pearson chi-square or Fisher’s exact test. The level of significance was set at a p-value of 0.05. All statistical analyses were performed using SPSS software version 16.0 for Windows (SPSS, Inc., Chicago, IL).

## Results

### Reperfusion Characteristics

**Figure 1:**
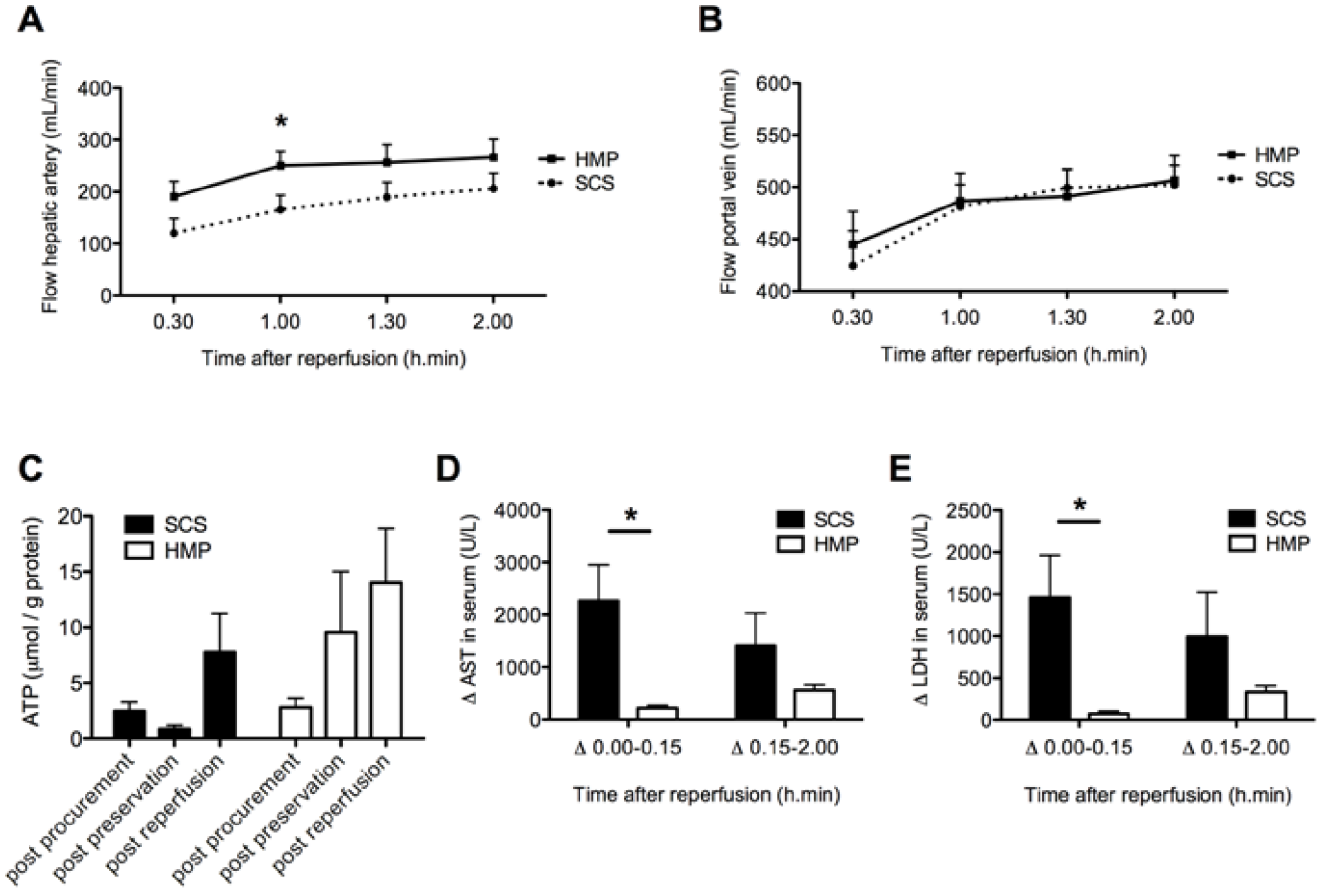
Perfusion characteristics during and changes in serum concentrations of AST and LDH after 2 h of normothermic *ex vivo* sanguineous reperfusion of DCD livers that were preserved by either 4 h of oxygenated HMP or SCS. *Panel A*: Blood flow through the hepatic artery. *Panel B*: Blood flow through the portal vein. *Panel C*: Changes in hepatic energy content as reflected by hepatic ATP content. *Panel D and E*: Relative increase of liver enzymes was significantly greater for SCS preserved livers, compared to livers preserved by oxygenated HMP. *p-value <0.05.

After 4 h of either HMP or SCS livers were reperfused *ex vivo* for 2 h at 37°C using oxygenated autologous blood. During the entire reperfusion period hepatic artery flow was higher in the HMP group, which was only statistically significant at 1 h after reperfusion (251±28 vs 166±28 mL/min; p = 0.003; **Figure 1A**). Portal vein flow was similar in the two groups (**Figure 1B**).

Cellular energy status as assessed by hepatic ATP content decreased during SCS (from 2.5±0.8 after procurement to 0.9±0.3 µmol/g protein after SCS; p = 0.048), but increased during oxygenated HMP (from 2.8±0.9 after procurement to 9.5±5.5 µmol/g protein after HMP). In both groups hepatic ATP content increased after normothermic reperfusion (7.8±3.5 and 14.0±4.9 µmol/g protein in the SCS and HMP groups, respectively; **Figure 1C**).

During reperfusion, no differences were observed in oxygenation of SCS versus HMP preserved livers; at 2 hours of reperfusion the sO_2_ was 98.0±0.0 vs 98.3±0.0% and the pO_2_ was 29.3±5.1 vs 30.8±4.4mmHg, respectively.

Mean weight of livers before and after HMP+reperfusion was 1427±208 g and 1381±154 g, respectively. Liver weight before and after SCS+reperfusion was 1301±207 g and 1610±161 g, respectively. Changes in weight were not significantly different between the two groups.

### Impact of HMP on Hepatocellular Injury and Function

Release of liver enzymes after reperfusion was used to assess the degree of hepatocellular injury. To correct for a wash out effect of machine perfusion, the relative increase in plasma levels of AST and LDH during and after the first 15 min of reperfusion was determined. For both time intervals increase in serum enzymes was lower in the HMP group, which was significant for the first time period (ΔAST 2264±687 vs 218±50 U/L, p = 0.008 and ΔLDH 1458±508 vs 73±31 U/L, p = 0.008; **Figure 1D–E**). Delta AST and ΔLDH plasma levels at 15-minute intervals are presented in *Figure S2*.

**Figure 2:**
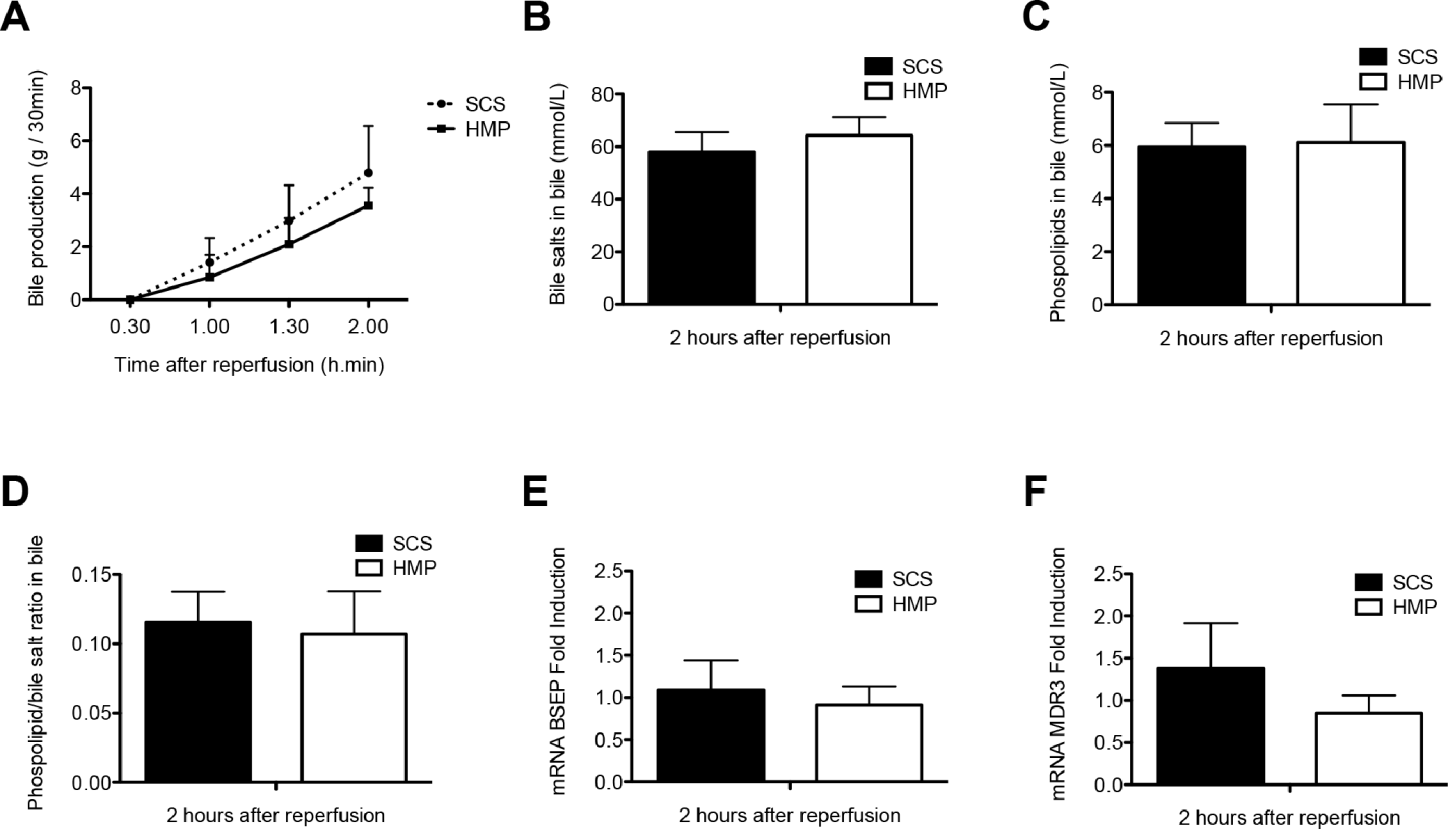
Parameters of hepatocellular secretory function of DCD livers that were preserved by either 4 h of oxygenated HMP or SCS and subsequently reperfused for 2 **h by normothermic** *ex vivo* sanguineous perfusion. *Panel A*: Evolution of bile production in the HMP and SCS group. *Panel B and C*: Biliary concentration of bile salts and phospholipids, respectively. *Panel D*: Bile salt toxicity, as represented by the ratio of biliary bile salt and phospholipid concentrations. There were no statistically significant differences between the groups. *Panel E–F*: Relative mRNA expression of the main hepatocellular bile transporters BSEP (bile salt export pump; Abcb11) and MDR3 (multidrug resistance protein 3, Abcb4) after 2 h of *ex vivo* sanguineous reperfusion of DCD livers that were preserved by either 4 h of oxygenated HMP or SCS. There were no significant differences between the two groups.

During reperfusion, increasing bile production was observed in both groups, without significant differences between the groups (**Figure 2A**). There were no significant differences in biliary concentrations of bile salts and phospholipids (SCS vs HMP: bile salts 58.0±7.5 vs 64.3±7.0 mmol/L, p = 1.000 and phospholipids 5.9±0.9 vs 6.1±1.4 mmol/L, p = 0.919, respectively), as well as their ratio at 2 h after reperfusion (SCS vs HMP: phospholipid/bile salt ratio 0.12±0.02 vs 0.11±0.03, p = 0.823) or in gene expression levels of the transporter proteins BSEP and MDR3, responsible for the biliary secretion of bile salts and phospholipids, respectively (BSEP fold induction 1.09±0.35 vs 0.91±0.22, p = 0.315 and MDR3 1.38±0.54 vs 0.85±0.21, p = 0.615 in SCS and HMP groups, respectively; **Figure 2B–F**).

### Impact of HMP on Biliary Epithelial Injury and Function

**Figure 3:**
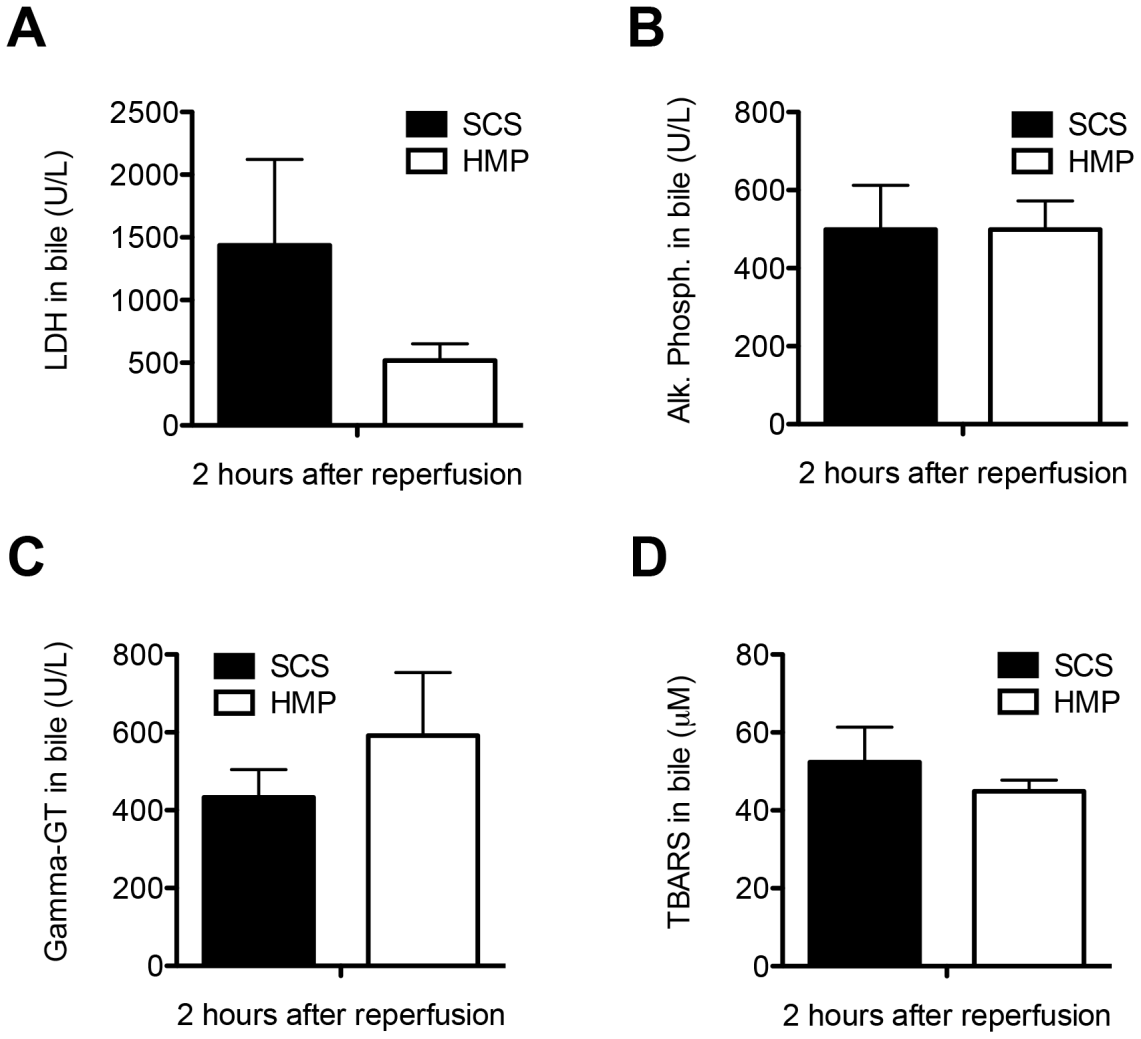
Comparison of biochemical parameters of bile duct injury and oxidative stress in DCD livers that were preserved by either 4 h of oxygenated HMP or 4 h of SCS and subsequently reperfused for 2 h by normothermic *ex vivo* sanguineous perfusion. *Panel A–C*: Concentration of LDH, alkaline phosphatase, and gamma-GT in bile samples at 2 h after reperfusion. *Panel D*: Comparison of biliary concentration of TBARS, a marker for oxidative stress and lipid peroxidation in bile ducts, at 2 h after graft reperfusion. There were no significant differences between the SCS preserved and HMP preserved livers.

**Figure 4:**
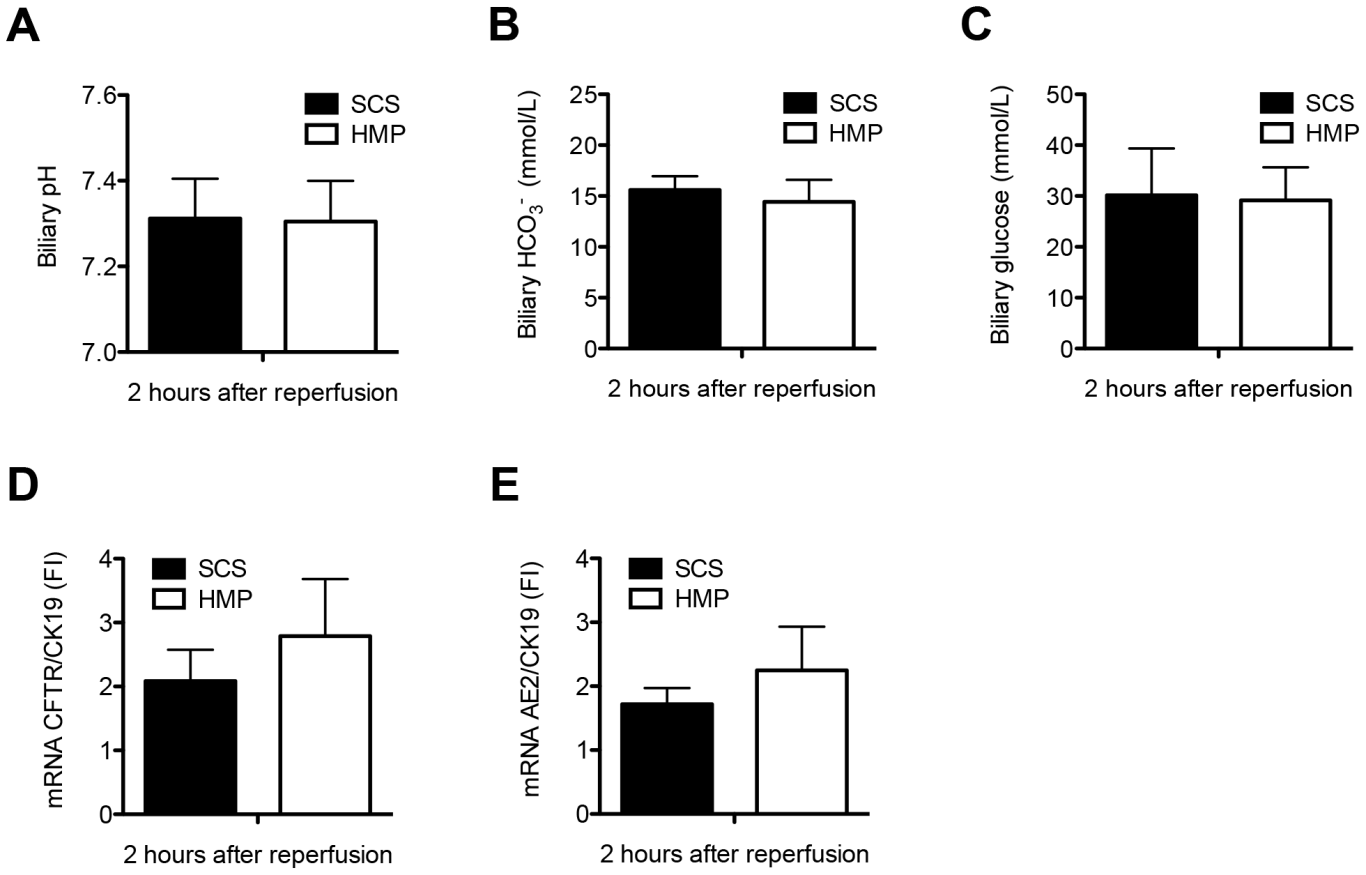
Comparison of functional parameters of biliary epithelial cell function in DCD livers that were preserved by either 4 h of oxygenated HMP or 4 h of SCS and subsequently reperfused for 2 h by normothermic *ex vivo* sanguineous perfusion. There were no significant differences in biliary pH (*Panel A*), biliary bicarbonate (*Panel B*) and glucose concentration (*Panel C*) at 2 h after reperfusion between the two groups. Relative mRNA expression of the main cholangiocyte transporter proteins involved in biliary bicarbonate secretion, CFTR (cystic fibrosis transmembrane conductance regulator; ABC35) (*Panel D*) and AE2 (anion exchanger 2; SLC4A2) (*Panel E*) after 2 h of *ex vivo* sanguineous reperfusion of DCD livers that were preserved by either 4 h of oxygenated HMP or SCS. There were no significant differences between the two groups.

Biliary concentrations of LDH, alkaline phosphatase and gamma-GT at 2 h after reperfusion were similar in the two groups, with LDH of 1436±685 vs 583±132 U/L (p = 1.000), alkaline phosphatase 499±114 vs 728±237 U/L (p = 1.000) and gamma-GT 433±71 vs 611±142 U/L (p = 0.283) in the SCS and HMP groups, respectively (**Figure 3A–C**). In parallel with this, there was no difference in concentration of TBARS in bile, a marker of oxidative stress in the bile ducts (SCS 52.4±9.0 µM vs HMP 44.9±2.9 µM; p = 0.454; **Figure 3D**). Moreover, there were no significant differences in biliary pH (SCS 7.3±0.1 vs HMP 7.3±0.1; p = 0.206) and concentrations of bicarbonate and glucose in bile at 2 h after reperfusion (SCS vs HMP: biliary bicarbonate 15.6±1.3 vs 14.9±1.7, p = 0.752 and glucose 30.1±9.2 vs 29.2±6.5, p = 0.932, respectively) or in gene expression of the biliary epithelial transporters involved in the secretion of bicarbonate into the bile, CFTR and AE2 between the two groups (SCS vs HMP: CFTR 2.09±0.49 vs 2.79±0.89, p = 0.504 and AE2 1.72±0.25 vs 2.25±0.68, p = 0.483, respectively; **Figure 4A–E**).

### Impact of HMP on Preservation of Hepatocellular and Biliary Morphology

**Figure 5:**
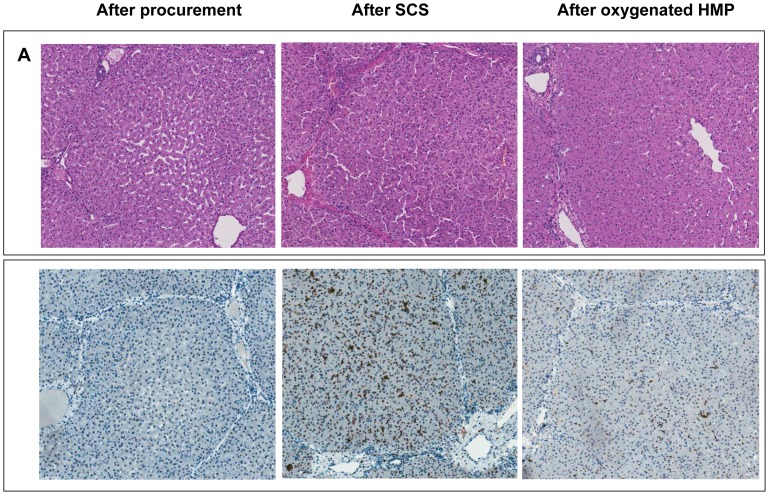
Representative examples of histology of liver parenchyma of DCD liver grafts preserved by either 4 h of SCS or 4 h of oxygenated HMP followed by 2 h by normothermic *ex vivo* sanguineous perfusion. *Panel A*: H&E staining of a central biopsy of the liver parenchyma. *Panel B*: Caspase-3 immunohistochemistry of liver parenchyma showing less intense caspase-3 staining of hepatocytes, sinusoidal endothelial cells, and Kupffer cells in the HMP group, compared to the SCS group. Brown color indicates immunopositivity. Original magnification 200x.

**Figure 6:**
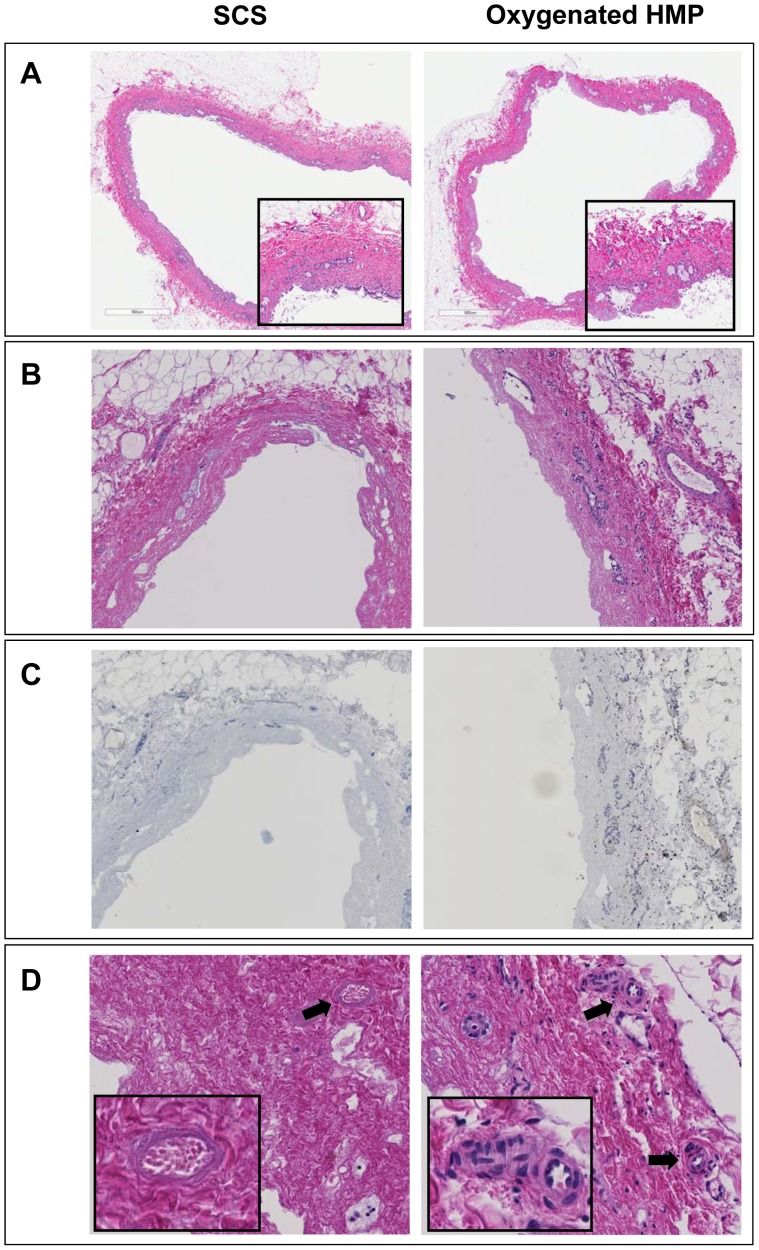
Representative examples of H&E histology of bile ducts of DCD liver grafts preserved by either 4 h of oxygenated HMP or 4 h of SCS followed by 2 h by normothermic *ex vivo* sanguineous perfusion. *Panel A*: extrahepatic bile duct immediately after procurement (magnification 200x). The insert represents a higher magnification of 400x. *Panel B*: extrahepatic bile duct after preservation and reperfusion (magnification 280x). *Panel C*: Immunohistochemistry for activated caspase-3 of the same bile ducts as presented in panel B (brown color indicates immunopositivity; counterstaining with hematoxylin). Very few caspase-3 positive cells were detected in the bile duct wall stroma. Remnant biliary epithelial cells (i.e. in the peribiliary glands) were not positive for acitivated caspase-3. *Panel D*: higher magnification (400x) of extrahepatic bile ducts focusing on the peribiliary plexus. Arrows indicate peribiliary arterioles. The insert represents a higher magnification of the peribiliary arterioles (800x). Bile ducts of livers preserved by oxygenated HMP displayed significantly less signs of arteriolonecrosis, compared to livers preserved by SCS (see also **Table 1**).

H&E staining and activated caspase-3 immunohistochemistry of liver parenchyma revealed less signs of ischemia-reperfusion injury in the HMP group, compared to SCS preserved livers (**Figure 5**). H&E staining of bile duct samples was used for semiquantitative analysis of bile duct injury, as described by Hansen et al [11]. The results of this morphological analysis of extrahepatic bile ducts are summarized in **Table 1**. In this model of DCD livers, we observed extensive loss of biliary epithelial lining immediately after organ procurement. The underlying mural stroma and peribiliary vascular plexus, however, appeared relatively normal (**Figure 6A**). After preservation and reperfusion, bile ducts of both HMP and SCS preserved livers displayed signs of increased mural necrosis with loss of cell nuclei in the subepithelial stroma (**Figure 6B**). Immunohistochemistry revealed very few activated caspase-3 positive cells, suggesting that cells died from necrosis rather than apoptosis (**Figure 6C**). Interestingly, the degree of arteriolonecrosis of the peribiliary vascular plexus was significantly lower in the HMP group, compared to the SCS group (>50% arteriolonecrosis was observed in 7 bile ducts of the SCS group versus 1 bile duct of the HMP group; p = 0.024; **Table 1** and **Figure 6D**).

**Table 1:** Histological analysis of bile duct injury according to Hansen et[11].

	Oxygenated HMP group	Static Cold Storage Group	p-value
	(n = 8)*	(n = 9)	
**Mucosal loss**			NA
Score 0 (no BEC loss)	0	0	
Score 1 (≤50% BEC loss)	0	0	
Score 2 (>50% BEC loss)	8	9	
**Mural necrosis**			0.915
Score 0 (no necrosis)	0	0	
Score 1 (≤25% necosis)	0	0	
Score 2 (25–50% necrosis)	1	1	
Score 3 (50–75% necrosis)	1	2	
Score 4 (>75% necrosis)	6	6	
**Arteriolonecrosis**			**0.024**
Score 0 (no arteriolonecrosis)	1	0	
Score 1 (≤50% arteriolonecosis)	6	2	
Score 2 (>50% arteriolonecrosis)	1	7	
**Bleeding**			NA
Score 0 (no bleeding)	8	9	
Score 1 (≤50% of the bile duct)	0	0	
Score 2 (>50% of the bile duct)	0	0	
**Thrombosis**			0.600
Score 0 (no thrombi)	7	7	
Score 1 (occurrence of thrombi)	1	2	

*Bile duct histology was missing for one pig in the HMP group. Abbreviations used: BEC, biliary epithelial cells; NA, not assessable.

## Discussion

Although several authors have proposed machine perfusion as an attractive alternative method for preservation of DCD livers, it remains to be established whether this technique will reduce the amount of bile duct injury and contributes to a reduction of biliary complications. We here report the first study on the impact of oxygenated HMP on the preservation of the bile ducts in a porcine DCD model. The most striking histological difference between oxygenated HMP and SCS was a lower degree of arteriolonecrosis of the peribiliary plexus in the extrahepatic bile ducts of grafts preserved by HMP. In parallel with this we observed a higher arterial blood flow after reperfusion of HMP preserved livers, compared to SCS. Although HMP did not result in a lower amount of injury of the biliary epithelial lining or underlying stroma, the observed difference in preservation of the peribiliary vascular plexus may be clinically relevant as the presence of arteriolonecrosis in extrahepatic bile duct biopsies at the time of transplantation was recently identified as the only independent histological parameter predictive for the development of NAS after human liver transplantation [11]. In the study by Hansen et al. a detailed scoring system was developed to study the morphology of bile ducts, which we applied in the current study to investigate our porcine donor bile ducts [11]. Machine perfusion of liver grafts may enable better preservation of the arterial vasculature of the biliary tree by providing a continuous supply of oxygen to the endothelium, better distribution of the preservation fluid, and wash out of waste products. Adequate blood supply to the bile ducts is known to be of critical importance in maintaining viability [20]. Insufficient arterial perfusion of liver grafts, either due to hepatic artery thrombosis or stenosis, leads to ischemic cholangiopathy, characterized by loss of biliary epithelium, necrosis of the bile duct wall and subsequent narrowing of the bile duct lumen due to sclerosing fibrosis [9], [21]. Therefore, better preservation of the peribiliary plexus and higher arterial flow after reperfusion as observed in the current study is an important finding.

Apart from the differences in arterial flow after reperfusion and better preservation of the peribiliary vascular plexus, we found no histological differences in amount of biliary epithelial cell loss or bile duct stroma. In parallel with this, biochemical parameters of biliary epithelial cell function (biliary pH and bicarbonate secretion) or biliary injury (concentrations LDH, AF, and gamma-GT in bile) did not differ between the two groups. Nevertheless, improved preservation and perfusion of the peribiliary vascular plexus as observed in the HMP group may result in faster and more efficient regeneration of the biliary epithelium. Two recent clinical studies have demonstrated that biliary epithelial cell loss can be found in more than 80% of all human liver grafts before transplantation [11], [22]. This interesting new finding has changed the perspective from which to view the pathogenesis of biliary strictures after transplantation as it suggests that the critical factor that determines whether a graft will develop biliary strictures or not is insufficient biliary regeneration rather than the initial biliary injury [23].

In accordance with previous studies [12], [16], [24], [25] we have observed a reduction in biochemical histological markers of hepatocellular injury and histological injury of the parenchyma. Release of ALT and LDH after graft reperfusion was lower in the HMP group, compared to SCS preserved livers. This difference in enzyme release during reperfusion can be explained to a large extent by the ‘wash out’ effect in HMP preserved livers, since those livers have been perfused with hypothermic preservation fluid for 4 h before reperfusion with porcine blood. Although preservation of the liver parenchyma was not the primary focus of the current study, these findings confirm previous studies suggesting that HMP provides better preservation of the liver parenchyma than SCS.

Although great progress has been made in the development of machine perfusion as an alternative to SCS for preservation of donor organs, it remains to be established which method of machine perfusion is most effective. Several variations of machine perfusion have been explored, including hypothermic oxygenated or non-oxygenated, subnormothemic and normothermic perfusion [12]–[13], [25]–[28]. It also remains unclear whether or not machine perfusion should replace SCS completely, or whether it is sufficient to provide a short period of machine perfusion at the end of cold storage before transplantation [16]. The most basic type of machine preservation is non-oxygenated hypothermic perfusion, a technique that was first applied in a clinical setting by Guarrera et al [12]. The advantage of this technique is its relative simplicity and safety, but there is doubt whether this method is sufficient to protect high-risk grafts such as those from DCD donors [24], [29]. Optimal preservation and protection of the biliary epithelium and bile duct wall may require a more advanced technique in which liver grafts are perfused with oxygenated perfusion fluid and at body temperature. Oxygenated HMP at 10°C, as used in the current study, has been successfully used in a recent study on end-ischemic oxygenated HMP in transplanted human DCD donor livers [30]. Using a porcine model of DCD livers, Boehnert et al recently reported favourable effects of normothermic acellular machine perfusion of bile duct preservation in pig DCD livers [31]. Our group previously demonstrated that normothermic, oxygenated perfusion of human donor livers is technically feasible; however, more research in this area is needed to determine whether this results in a reduction of biliary complications after transplantation [13].

In conclusion, this study suggests that oxygenated HMP of liver grafts from DCD donors provides protection against ischemic injury of the peribiliary vascular plexus, as reflected by a lower degree of arteriolonecrosis in the bile duct wall. Moreover, it associated with higher arterial flow rates early after reperfusion, compared to SCS. Although, we observed no differences in the degree of biliary epithelial cell loss or bile duct necrosis between livers preserved by HMP or SCS, the combination of better preservation of the peribiliary plexus and higher arterial flow may contribute to a faster recovery of the post ischemic bile ducts. Whether this results in a reduction of the rate of biliary complications after transplantation of DCD livers should be answered in clinical trials.

## Supporting Information

Figure S1 **Perfusion characteristics of DCD livers during 4 h of oxygenated hypothermic machine perfusion.** *Panel A*: Flow of perfusion fluid through the hepatic artery. *Panel B*: Flow of perfusion fluid through the portal vein.
(TIF)

Figure S2 **Changes in serum concentrations of AST and LDH during** ***ex vivo*** **sanguineous reperfusion of DCD livers that were preserved by either 4 h of oxygenated HMP or SCS.** *Panel A–B*: The relative increase of liver enzymes was significantly greater for SCS preserved livers, compared to livers preserved by oxygenated HMP, at all time points after the start of reperfusion. *p-value <0.05.
(TIFF)

File S1 **Supplementary digital content.** Information complimentary to the Materials and Methods section of this paper. Measurement of thiobarbituric acid reactive substances (TBARS), gene expression of hepatobiliary transporter proteins and ATP extraction and measurement are discussed here.
(DOC)

Table S1 **Sequences of primers used for real time RT PCR analysis are presented in this table.**
(DOC)
