# Supplementary figures and images for: Hypothermic Oxygenated Machine Perfusion Prevents Arteriolonecrosis of the Peribiliary Plexus in Pig Livers Donated after Circulatory Death
Source: PLoS One. 2014 Feb 14;9(2):e88521. doi: 10.1371/journal.pone.0088521 (PMC3925142; doi:10.1371/journal.pone.0088521)

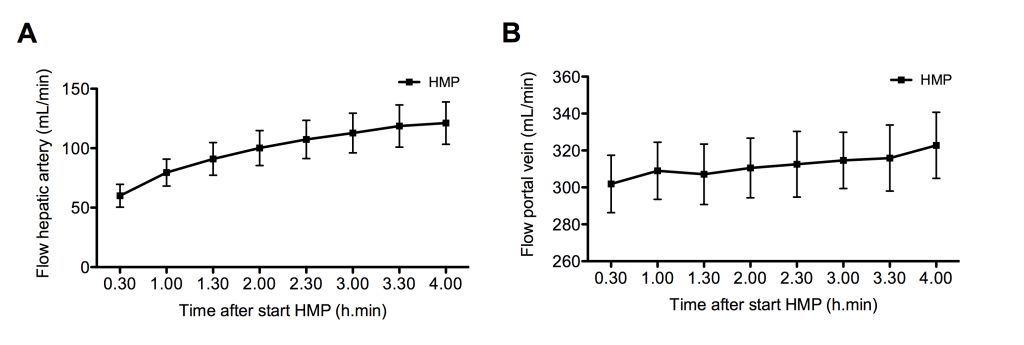

Supplement: Figure S1 — Perfusion characteristics of DCD livers during 4 h of oxygenated hypothermic machine perfusion. Panel A: Flow of perfusion fluid through the hepatic artery. Panel B: Flow of perfusion fluid through the portal vein. (TIF) [file pone.0088521.s001.tif]

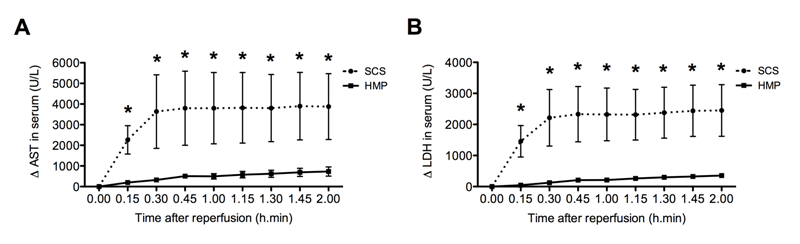

Supplement: Figure S2 — Changes in serum concentrations of AST and LDH during ex vivo sanguineous reperfusion of DCD livers that were preserved by either 4 h of oxygenated HMP or SCS. Panel A–B: The relative increase of liver enzymes was significantly greater for SCS preserved livers, compared to livers preserved by oxygenated HMP, at all time points after the start of reperfusion. *p-value <0.05. (TIFF) [file pone.0088521.s002.tiff]
