# Supplementary material for: Hypothermic Oxygenated Machine Perfusion Prevents Arteriolonecrosis of the Peribiliary Plexus in Pig Livers Donated after Circulatory Death
Source: PLoS One. 2014 Feb 14;9(2):e88521. doi: 10.1371/journal.pone.0088521 (PMC3925142; doi:10.1371/journal.pone.0088521)
Supplement: File S1 — Supplementary digital content. Information complimentary to the Materials and Methods section of this paper. Measurement of thiobarbituric acid reactive substances (TBARS), gene expression of hepatobiliary transporter proteins and ATP extraction and measurement are discussed here. (DOC) [file pone.0088521.s003.doc]

**SupplementaRY Digital Content**

**Measurement of thiobarbituric acid reactive substances (TBARS)**

TBARS were measured in bile samples as a marker for oxidative stress in bile ducts (see Supplemental Digital Ccontent). Twenty microliters of bile (1:10 diluted with H_2_O) was mixed with 90μL sodium dodecyl sulfate, 10μL 0.05M butylated hydroxytoluene, 400μL 0.1 N HCL, 50μL 10% phosphotungstic acid and 200μL 0.7% 2-thiobarbituric acid. Samples were kept at 92-97°C for exactly 30 min and the content was mixed with 800μL 1-butanol. After centrifugation for 10 min (3,000 rpm/960g), samples from the butanol layer were transferred to a 96-well microtitre plate and fluorescence was detected at an excitation wavelength of 530nm and emission wavelength of 590nm. TBARS concentrations were calculated from a calibration curve of malondialdehyde constructed on the same plate.

***Gene Expression of Hepatobiliary Transporter Proteins***

Hepatic mRNA expression of relevant hepatocellular and cholangiocyte transporter proteins involved in bile secretion was determined by quantitative real-time PCR. Total RNA was isolated from frozen liver tissue using TRIzol (Gibco Life Techologies, Grand Island, NY), and M-MLV reverse transcriptase (Invitrogen, Basel, Switzerland) was used to convert RNA into cDNA. For quantitative real-time detection, sense and antisense porcine-specific primers (Invitrogen, Paisly, Scotland) were designed for the following genes, using Primer Express software (Applied Biosystems, Foster City, CA): the hepatocellular transporters BSEP (bile salt export pump; Abcb11) and MDR3 (the phospholipid translocator multidrug resistant protein 3; Abcb4), as well as the biliary epithelial transporters involved in biliary bicarbonate secretion CFTR (cystic fibrosis transmembrane conductance regulator; Abc35) and AE2 (anion exchanger 2; Slc4a2), CK19 (cytokeratin 19; used as internal standard for biliary epithelium) and 18s rRNA (internal standard). Amplification and detection were performed with the ABI Prism 7900HT Sequence Detection System using emission from SYBR® Green (Applied Biosystems). Copy numbers of CK19 and transporter gene mRNA were normalized for 18s rRNA and additionally for CK19 mRNA if appropriate.

***Adenosine-5'-triphosphate (ATP) Extraction and Measurement***

Hepatic concentration of ATP was used as an indicator of the energy status of grafts. Liver samples were immediately frozen in liquid nitrogen. Frozen tissue was cut into 20μm slices and a total amount of ±50mg was homogenized in 1 mL of SONOP (0.372g EDTA in 130mL H_2_O and NaOH (pH 10.9) + 370 mL 96% ethanol) and sonificated. Precipitate was removed by centrifugation (13,000 rcf for 10 min). Supernatant was diluted with SONOP to attain a protein concentration of 200-300 μg/mL (Pierce BCA Protein Assay Kit, Thermo Scientific, Rockford, IL) and mixed with 450μL of 100mM phosphate buffer (Merck; pH 7.6-8.0). Fifty microliters of phosphate buffered supernatant was used for ATP measurement using ATP Bioluminescence assay kit CLS II (Boehringer, Mannheim, Germany) and a luminometer (Victor^3TM^ 1420 multilabel counter, PerkinElmer). ATP concentrations were calculated from a calibration curve constructed on the same plate, corrected for amount of protein, and values were expressed as µmol/g protein.
