# Supplementary material for: Hypothermic Oxygenated Machine Perfusion Prevents Arteriolonecrosis of the Peribiliary Plexus in Pig Livers Donated after Circulatory Death
Source: PLoS One. 2014 Feb 14;9(2):e88521. doi: 10.1371/journal.pone.0088521 (PMC3925142; doi:10.1371/journal.pone.0088521)
Supplement: Table S1 — Sequences of primers used for real time RT PCR analysis are presented in this table. (DOC) [file pone.0088521.s004.doc]

**Supplementary Table S1. Sequences of primers used for real time RT PCR analysis**

| Gene | Code |  | Primers | PCR product (bp) |
| --- | --- | --- | --- | --- |
| BSEP  (Abcb11) | DQ530510 U20587 | Forward | 5’-GCC AAG AAA GGA GCC TAC TAC AAA C-3’ | 79 |
|  |  | Reverse | 5’-CTG TGC ATG AAG TTC TCA AGT CAG AT-3’ |  |
| MDR3  (Abcb4) | EF067318 | Forward | 5’-CCA GGA AGC AAA GAA ACT CAA TG-3’ | 139 |
|  |  | Reverse | 5’-CTC CTC CAG GGT CAC AAT GC-3’ |  |
| CFTR (ABc35) | NM_ 001104950.1 | Forward | 5’-AATGACTGTCAAAGACCTCACTGC-3’ | 55 |
|  |  | Reverse | 5’-TAATACGGCGTTCCCACCAT-3’ |  |
| AE2 (Slc4a2) | XM_003360081.2 | Forward | 5’-GATTTTCCTGTACATGGGTGTCAC-3’ | 53 |
|  |  | Reverse | 5’-GCTCGTAGAACTGGATTCCGTTAA-3’ |  |
| CK19 | XM_003131437.1 | Forward | 5’-TGCCACCATTGAGAACTCCA-3’ | 52 |
|  |  | Reverse | 5’-CAGACGGGCGTTGTCGA-3’ |  |
| 18s | NR_46261 | Forward | 5’-AGTCCCTGCCCTTTGTACACAC-3’ | 51 |
|  |  | Reverse | 5’-AACCATCCAATCGGTAGTAGCG-3’ |  |
